# Unified treatment algorithm for the management of crotaline snakebite in the United States: results of an evidence-informed consensus workshop

**DOI:** 10.1186/1471-227X-11-2

**Published:** 2011-02-03

**Authors:** Eric J Lavonas, Anne-Michelle Ruha, William Banner, Vikhyat Bebarta, Jeffrey N Bernstein, Sean P Bush, William P Kerns, William H Richardson, Steven A Seifert, David A Tanen, Steve C Curry, Richard C Dart

**Affiliations:** 1 Rocky Mountain Poison and Drug Center, Denver Health and Hospital Authority, Denver, Colorado, USA; 2 Division of Medical Toxicology, Department of Emergency Medicine, University of Colorado School of Medicine, Aurora, Colorado, USA; 3 Department of Medical Toxicology, Banner Good Samaritan Medical Center, Phoenix, Arizona, USA; 4 Oklahoma Poison Center, College of Pharmacy, University of Oklahoma, Oklahoma City, Oklahoma, USA; 5 Pediatric Intensive Care Unit, Integris Baptist Medical Center, Oklahoma City, Oklahoma, USA; 6 Department of Emergency Medicine, Wilford Hall Medical Center, San Antonio, Texas, USA; 7 Florida Poison Information Center, Miami, Florida, USA; 8 Emergency Care Center, Jackson Memorial Hospital, Miami, Florida, USA; 9 Department of Emergency Medicine, Loma Linda University School of Medicine, Loma Linda, California USA; 10 Division of Medical Toxicology, Department of Emergency Medicine and Carolinas Poison Center, Carolinas Medical Center, Charlotte, North Carolina, USA; 11 Department of Emergency Medicine, Palmetto Health Richland, Columbia, South Carolina, USA; 12 Palmetto Poison Center, University of South Carolina, Columbia, South Carolina, USA; 13 New Mexico Poison and Drug Information Center, College of Pharmacy, University of New Mexico, Albuquerque, New Mexico, USA; 14 Department of Emergency Medicine, University of New Mexico School of Medicine, Albuquerque, New Mexico, USA; 15 Naval Medical Center, San Diego, California, USA; 16 Division of Medical Toxicology, Department of Emergency Medicine, University of California at San Diego, San Diego, California, USA

## Abstract

**Background:**

Envenomation by crotaline snakes (rattlesnake, cottonmouth, copperhead) is a complex, potentially lethal condition affecting thousands of people in the United States each year. Treatment of crotaline envenomation is not standardized, and significant variation in practice exists.

**Methods:**

A geographically diverse panel of experts was convened for the purpose of deriving an evidence-informed unified treatment algorithm. Research staff analyzed the extant medical literature and performed targeted analyses of existing databases to inform specific clinical decisions. A trained external facilitator used modified Delphi and structured consensus methodology to achieve consensus on the final treatment algorithm.

**Results:**

A unified treatment algorithm was produced and endorsed by all nine expert panel members. This algorithm provides guidance about clinical and laboratory observations, indications for and dosing of antivenom, adjunctive therapies, post-stabilization care, and management of complications from envenomation and therapy.

**Conclusions:**

Clinical manifestations and ideal treatment of crotaline snakebite differ greatly, and can result in severe complications. Using a modified Delphi method, we provide evidence-informed treatment guidelines in an attempt to reduce variation in care and possibly improve clinical outcomes.

## Background

Envenomation by pit vipers (family *Viperidae*, subfamily *Crotalinae*, genera *Crotalus, Agkistrodon, and Sistrurus*) is a dynamic and potentially serious medical condition. Approximately 9,000 patients are treated for snakebite and 5 die in the United States (US) each year [1,2]. The use of antivenom is increasing over time. Forty-four percent of patients whose cases were reported to US poison centers in 2007 were treated with antivenom, a significant increase from 30% in 2000 [3]. The proportion of patients receiving antivenom varies more than 5-fold between states. Poison center data suggest a case-fatality rate among rattlesnake victims of approximately 1 death per 736 patients [4].

The clinical manifestations of crotaline envenomation vary considerably based on a complex interplay between the victim and the venom exposure. Some critical manifestations, such as airway involvement and anaphylaxis to venom, are so uncommon that few clinicians gain experience managing these findings. To our knowledge, all extant treatment algorithms were created by a single author or by a small group of authors with similar experience [5-8]. Many algorithms are specific for the treatment of subpopulations of crotaline victims, such as children or those envenomated in regions where copperhead snakes predominate. Few authors describe their methods for algorithm development, and many algorithms do not fully describe post-stabilization care. Significant variations in practice exist; two studies demonstrate that the proportion of snakebite victims who undergo fasciotomy is five times greater in an institution where snakebite victims are managed primarily by surgeons, compared to an institution where snakebite victims are admitted and managed primarily by medical toxicologists [9,10]. Antivenom is expensive (current wholesale cost greatly exceeds US$1,000/vial) and associated with immunologic risk, and it is imperative for the physician to use this resource wisely. The objective of this project was to produce an evidence-informed unified treatment algorithm for pit viper snakebite management in the US, with the goal of reducing unnecessary variations in practice and improving outcomes for snake envenomation victims.

## Methods

Because only one randomized clinical trial involving the treatment of crotaline snakebite with antivenom has ever been published, a formal meta-analysis could not be used for rule development [11]. A standardized evidence-based rule development process, such as that proposed by the GRADE working group, cannot be used to develop an algorithm because the clinical questions cannot be defined in advance. Therefore, using a trained external facilitator, we used structured methods to achieve an evidence-informed consensus among a diverse group of experts.

Two authors (EJL, RCD) recruited panel members based on their published envenomations research and clinical experience. In order to ensure a diversity of experience, panel members were chosen from across the regions of the US where crotaline envenomations are common, with no more than one panel member chosen from the same geographic area. A group size of nine experts was chosen to permit the required diversity of experience while keeping the consensus-building process manageable. One of the original panel members (SCC) had to withdraw from the process; he was replaced on the panel by a colleague from the same institution, but remained involved in the project as a non-voting participant and contributor. The nine panel members have extensive clinical experience managing crotaline snakebite in a variety of clinical settings (Table 1), and have published 57 peer-reviewed articles on the subject. One additional author (EJL) participated in the panel meeting but did not vote.

**Table 1:** Panel Member Qualifications

Panel Member	Board Certification	Practice Setting	Practice Location
William Banner, MD, PhD	Pediatrics, pediatric critical care, medical toxicology	Clinical toxicology service, pediatric intensive care unit	Oklahoma City, Oklahoma, USA

Vikhyat Bebarta, MD	Emergency medicine, medical toxicology	Clinical toxicology service, emergency department	San Antonio, Texas, USA

Jeffrey Bernstein, MD	Emergency medicine, medical toxicology, clinical pharmacology	Emergency department, poison center	Miami, Florida, USA

Sean P. Bush, MD	Emergency medicine	Envenomations clinical service, emergency department	Loma Linda, California, USA

Richard C. Dart, MD	Emergency medicine, medical toxicology	Clinical toxicology service, poison center	Denver, Colorado, USA

William P. Kerns, II, MD	Emergency medicine, medical toxicology	Clinical toxicology service, emergency department, poison center	Charlotte, North Carolina, USA

William H. Richardson, MD	Emergency medicine, medical toxicology	Emergency department, poison center	Columbia, South Carolina, USA

Anne-Michelle Ruha, MD	Emergency medicine, medical toxicology	Clinical toxicology service, emergency department, poison center	Phoenix, Arizona, USA

Steven A. Seifert, MD	Emergency medicine, medical toxicology	Clinical toxicology service, emergency department, poison center	Albuquerque, New Mexico, USA

David A. Tanen, MD	Emergency medicine, medical toxicology	Clinical toxicology service, emergency department, poison center	San Diego, California, USA

The consensus process was managed by a professional facilitator (David Kovick, JD, Consensus Building Institute, Cambridge, MA). Competing interests of all participants were disclosed prior to decision-making. One author (EJL) created an initial "straw man" draft algorithm, which was distributed to all panelists. The draft algorithm identified key decision points in the treatment process, posed questions about best treatment practices, and served as a starting point for discussion. Initial modifications to the "straw man" were processed using a modified Delphi methodology, through which panelists provided substantive feedback through the facilitator. The revised algorithm was presented to the panel in a 90-minute webinar, where facilitated discussion was used to identify initial areas of consensus and prioritize issues requiring further discussion. A second round of modified Delphi revisions was then completed. Final algorithm development took place during a 1.5-day in-person meeting held in Denver, Colorado, in May, 2010, which was governed by a structured consensus-building process. In resolving points of divergence among panel members, the panel relied upon published data (where available), supported by the collective experience of panel members. Consensus was defined as unanimous agreement of all panel members. After minor text revisions, the final algorithm was sent to panelists electronically for a conclusive vote.

In order to provide the panel members with a complete literature base, research staff performed a structured literature search to identify articles relevant to the treatment of crotaline snakebite in the United States, using the search strategy in Table 2. Two researchers reviewed the titles and abstracts of all articles to identify those which might contain original data about (a) the management of crotaline snakebite with the current (ovine Fab) antivenom or (b) the management of crotaline snakebite without antivenom. In the event of disagreement, the article was pulled and reviewed. Full text copies of the 42 articles containing original data relevant to the key questions identified in preliminary panel deliberations were obtained and provided for panel members' use during deliberations.

**Table 2:** Search Strategy

Database	Pub Med	Ovid Medline	EMBASE
Dates searched	1/1/1990 - 12/31/2009	1/1/1990 - 12/31/2009	1990 - 2009

Search terms employed (all connected by logical "OR" function)	MeSH headings:	MeSH headings:	
	Crotalid venoms/PO [poisoning]	Crotalid venoms/PO [poisoning]	Crotalid venoms AND [intoxication OR toxicity]
	Crotalid venoms/TO [toxicity]	Crotalid venoms/TO [toxicity]	Snake venoms AND [intoxication OR toxicity]
	Snake venoms/PO	Snake venoms/PO	Snake bites AND [drug therapy OR therapy]
	Snake venoms/TO	Snake venoms/TO	
	Snake bites/DT [drug therapy]	Snake bites/DT [drug therapy]	Viperidae
	Snake bites/TH [therapy]	Snake bites/TH [therapy]	Agkistrodon
	Viperidae	Viperidae	Crotalus
	Agkistrodon	Agkistrodon	FabAV
	Crotalus	Crotalus	Crotaline immune Fab
	Keywords	Keywords	
	CroFab	CroFab	
	Crotaline immune Fab	Crotaline immune Fab	

Citations retrieved	1230	1097	1711

Searches were conducted on January 6, 2010 and were limited to English language and humans. After removal of 1,748 duplicate citations, 339 additional citations were excluded based on the keywords rat(s), mouse, mice, rabbit(s), cellular, *in vivo*, or *in vitro*. Hand-search of the titles and abstracts of the remaining 1,951 citations yielded 91 citations that appeared to contain original data about crotaline snake envenomation patients who were either treated with Fab antivenom or managed without antivenom. Full-text copies of these 91 articles and abstracts were obtained and made available to the project team in a computer data file. Of these, 42 articles and abstracts were identified as containing data relevant to the key questions identified in preliminary panel discussions. These 42 references were reproduced and made available during the in-person panel meeting.

Recurrence of one or more venom effects (local pain and swelling and/or hematologic abnormalities such as coagulopathy and thrombocytopenia) following successful initial treatment with antivenom is a known problem in the management of venomous snakebite. Early issue identification revealed that prevention and treatment of these recurrence phenomena was a topic with some disagreement. Four data sources were utilized to inform the panel discussion of this issue. Statisticians reanalyzed raw data from databases created in the premarketing studies of the current antivenom to extract specific information about recurrence phenomena [11,12]. The same statistical team reanalyzed raw data from databases created in a phase IV post-marketing study of Fab antivenom to extract specific information about recurrence phenomena [13]. The research team reviewed the results of the literature search to identify and summarize all articles containing data about recurrence phenomena. These three data sources were prepared into resource documents for the panel members. During the in-person meeting, two authors provided formal presentations. One panelist (AMR) analyzed and presented case-level data about recurrence phenomena observed at her center, while a second participant (EJL) presented a structured review of the literature related to recurrence phenomena. In addition, three panelists provided informal presentations. One panelist (SAS) presented an analysis of the prognostic significance of fibrin split products in the identification of patients at risk for late hematologic effects, while two other panelists (SPB and WB) presented data about recurrence phenomena at their centers.

### Role of the funding source

This was an investigator-initiated project conceived, designed, and executed by two authors (EJL and RCD) and other Rocky Mountain Poison and Drug Center staff. The antivenom manufacturer provided funding support. Sponsor representatives were not present during the webinar or panel discussions. Sponsor representatives reviewed the final manuscript before publication for the sole purpose of identifying proprietary information. No modifications of the manuscript were requested by the manufacturer.

## Results

### Final unified treatment algorithm

**Figure 1:**
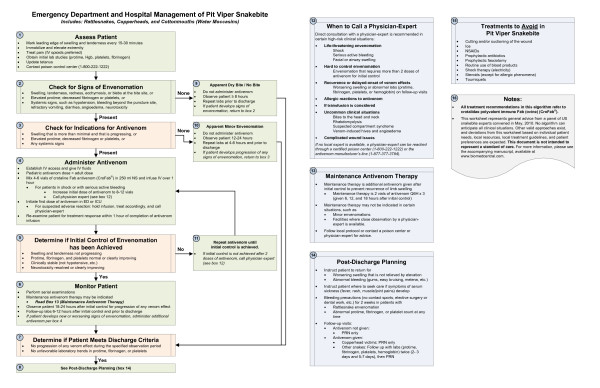
**Unified Treatment Algorithm for the Management of Pit Viper Snakebite in the United States**.

The unified treatment algorithm is shown in Figure 1. The final version was endorsed unanimously. Specific considerations endorsed by the panelists are as follows:

### Role of the unified treatment algorithm (general considerations and box 16)

This algorithm pertains to the treatment of human patients bitten by pit viper snakes (family *Viperidae*, subfamily *Crotalinae*) in the US, including the rattlesnakes (genus *Crotalus*), pygmy rattlesnakes (*Sistrurus*), and moccasin snakes (genus *Agkistrodon*). Within the *Agkistrodon *genus are the copperhead snakes (*A. contortrix*) and the water moccasin (cottonmouth) snake (*A. piscivorus*). This algorithm does not apply to treatment of patients bitten by coral snakes (family *Elapidae*), nor by snakes that are not indigenous to the US.

At the time this algorithm was developed, the only antivenom commercially available for the treatment of pit viper envenomation in the US is Crotalidae Polyvalent Immune Fab (ovine) (CroFab^®^, Protherics, Nashville, TN). All treatment recommendations and dosing apply to this antivenom. This algorithm does not consider treatment with whole IgG antivenom (Antivenin (Crotalidae) Polyvalent, equine origin (Wyeth-Ayerst, Marietta, Pennsylvania, USA)), because production of that antivenom has been discontinued and all extant lots have expired. This antivenom also does not consider treatment with other antivenom products under development. Because the panel members are all hospital-based physicians, the panel did not evaluate field first aid or other prehospital therapy.

In order to create an algorithm that was simple enough to be used effectively, the panel decided not to include specific recommendations for the management of certain rare manifestations of crotaline snakebite. These included snakebites to the head and neck, snakebites causing rhabdomyolysis, and apparent anaphylactic or anaphylactoid reactions to venom. In addition the panel recognized that no treatment algorithm could provide ideal advice for all situations or serve as a substitute for clinical judgment. Legitimate variations in practice will always exist, and care may appropriately vary based on several factors, including patient presentation, available treatment resources, patient comorbidities, and patient preference. The panel explicitly determined that the consensus treatment algorithm is not a standard of care.

### Patient assessment and initial management (box 1)

The initial approach to management of a patient with suspected pit viper snake envenomation begins with history, physical examination, and measurement of vital signs. Palpation of the envenomated area and marking the leading edge of swelling and tenderness every 15 - 30 minutes is a useful way to determine whether local tissue effects have stabilized or are progressing [7]. Although not evidence based, the panel recommends immobilization and elevation of the envenomated extremity to reduce swelling. In order to avoid obstructing lymphatic outflow, speed resolution of swelling, and possibly reduce the risk of blister formation in flexor creases, major joints such as the elbow should be maintained in relative extension (≤ 45 degrees of flexion).

Opioids are preferred over non-steroidal anti-inflammatory drugs (NSAIDs) because of the theoretical risk of bleeding associated with NSAID use in patients who may develop coagulopathy or thrombocytopenia due to envenomation. Although *Clostridium tetani *infection has not been reported following crotaline snakebite, it has occurred following envenomation by other vipers [14,15]. Standard recommendations for tetanus booster immunization (DTaP, Tdap, or Td as appropriate for the patient's age) should be followed [16].

Notification of a certified poison center is recommended for all cases of snake envenomation, for two reasons. First, poison center personnel can identify situations where use of this algorithm may be inappropriate, and can provide treatment recommendations based on local snake species and medical treatment resources. Second, certified poison centers provide de-identified data to the National Poison Data System, which is used by public health professionals and policy-makers. In the US, access to a certified poison center can be made through a single, toll-free number: 1-800-222-1222.

### Signs of crotaline envenomation (box 2)

Approximately 80% of pit viper bites result in the injection of venom [17,18]. Pit viper venom is a complex mixture of proteins and other macromolecules, with more than 50 identified components. The clinical effects produced by envenomation can be broadly classified into three groups. *Local tissue effects *include soft tissue necrosis and chemically mediated inflammation. A number of venom components, including myotoxic phospholipases A2 such as crotoxin, venom metalloproteinases that activate tumor necrosis factor-alpha (TNF-α), myotoxin *a*, hyaluronidase, phosphomonoesterase, phosphodiesterase, arginine ester hydrolase, and histamine- and bradykinin-like factors, cause direct tissue injury and produce a broad cytokine response in the victim [7,19-22]. Clinically, these effects are evident as pain, redness, swelling, tenderness, and myonecrosis that begin adjacent to the bite site and spread with movement of the venom through the lymphatic system. More than 90% of envenomated pit viper victims develop local tissue effects [7]. *Hematologic venom effects *include fibrinogen degradation and platelet aggregation and destruction [23,24]. On a laboratory basis, these are manifest by decreased fibrinogen levels, elevated prothrombin time, and thrombocytopenia. Detection of fibrin split products may be an early sign of a hematologic venom effect, and is a sensitive predictor of subsequent coagulopathy. In prospective studies, the presence of fibrin split products within the first 12 hours of treatment predicted subsequent hypofibrinogenemia with 87% sensitivity and 69% specificity [25]. In some patients, elevated fibrin split products were the only early signs of developing hypofibrinogenemia. Clinically, oozing of blood from the bite site and ecchymosis of the surrounding tissue are common. Systemic bleeding may manifest as nuisance bleeding, such as gingival bleeding or haemolacria, or more serious bleeding, such as significant epistaxis, gastrointestinal bleeding, or intracranial hemorrhage. Even among the population with severe defibrination or thrombocytopenia, most patients do not develop medically significant bleeding [26]. However, severe and fatal bleeding complications have been reported [27-31]. *Systemic venom effects *include hypotension from direct cardiovascular toxicity, third-spacing and vasodilatation, nausea and vomiting, angioedema, and neurotoxicity. Many pit vipers envenomations can cause patients to experience a metallic taste and localized neuromuscular effects (fasciculation and myokymia). Severe systemic neurotoxicity induced by Mojave toxin A, including cranial neuropathy and flaccid paralysis, are frequent manifestations from Mojave rattlesnake (*Crotalus scutulatus*) and Southern Pacific rattlesnake (*C. helleri*) envenomation, but have been rarely reported following envenomation by other US rattlesnake species [32-34]. Even within the same species, significant regional variations exist in neurotoxic venom components [35].

In practice, the treating physician can assess for all of these venom effects with a focused history and physical examination and review of basic laboratory studies. Serial measurements of prothrombin time, hemoglobin, and platelet counts are recommended for all pit viper victims. Fibrinogen is a more sensitive measure of venom-induced defibrination than prothrombin time, and should be followed, if obtainable. Although one-time measurement of fibrin split products in the first 12 hours post-bite is useful for early detection of incipient hematologic venom effects, no proven role in therapy has been established for serial fibrin split product measurements, and an elevated FSP alone is not an indication for antivenom treatment [25].

Most treatment resources include a grading scale for crotaline envenomation. The reliability and validity of these scales have not been established. Furthermore, because snake envenomation is a dynamic disease state, grading assigned at a single point in time may be a poor representation of overall severity. The panel members unanimously concluded that these scales are of little value outside of a research context, and therefore did not include a grading scale in these recommendations. Instead, the panel recommends serial examination of the patient for specific venom effects, with treatment based on the evolution of medically significant venom effects over time.

### Indications for antivenom (box 3)

Administration of antivenom, in adequate doses, effectively halts the spread of local tissue effects, reduces hematologic venom effects, and reduces systemic effects resulting from crotaline envenomation [11,12,26,36,37]. Treatment with antivenom is indicated for any patient with progressive local tissue effects, hematologic venom effects, and systemic signs attributable to venom. The panel recommends withholding antivenom from patients with limb envenomations who have localized pain and swelling as the only manifestation of envenomation, provided that these local tissue effects are not progressing. For extremity envenomations, some panelists use a threshold of swelling that has crossed a major joint [wrist, elbow, ankle, or knee] and is progressing for this purpose, while other panelists treat minor hand envenomations more aggressively. Unfortunately, it is not known whether early administration of antivenom in a patient with apparently minor envenomation improves long-term limb functional outcomes [38]. Regardless of the threshold chosen, patients with apparently minor envenomations require close observation, and should be given antivenom promptly if venom effects are progressing.

Because hematologic venom effects can progress over time, all patients seen early after envenomation with significantly abnormal prothrombin time, fibrinogen, and/or platelet count caused by envenomation should receive antivenom. Patients with hypotension, systemic bleeding, or other systemic venom effects should receive antivenom emergently. Any degree of true neurotoxicity, including localized fasciculations or myokymia, is an indication for antivenom administration. Some patients may present with symptoms attributable to anxiety; in the absence of signs of progressive envenomation, these patients can be reassured and observed.

### Antivenom administration (box 4)

Antivenom dosing is titrated to clinical response. The targeted clinical response is often termed, "initial control of the envenomation syndrome," and consists of arrest of the progression of local tissue venom effects, a clear trend toward improvement in any hematologic venom effects, and resolution of all systemic venom effects (excluding fasciculations or myokymia, which may be refractory to antivenom [7,11]. An initial dose of 4 to 6 vials was chosen for the premarketing trials because of equivalent binding capacity to then-standard doses of equine antivenom and was shown to be effective in two premarketing studies [11,12]. Subsequent experience has shown that most victims of rattlesnake envenomation achieve initial control with one or two such doses, while most copperhead snake victims can be successfully treated with a single 4-vial dose [39,40]. Very few patients continue to experience progressive venom effects after 18 vials of antivenom [36,41]. However, with the exception of a single case report, patients who did not achieve initial control after 20 vials of antivenom do not respond to subsequent doses [26,29,30,36]. Panel members noted that inexperienced health care providers sometimes use large doses of antivenom in an attempt to treat clinical effects that did not respond to therapy, but could be safely observed. The reason for limiting initial dosing to 4 to 6 vials is primarily cost, but also the theoretical increased risk of serum sickness with larger protein loads. Initial control doses of less than 4 vials have not been well studied.

Antivenom should be administered via intravenous infusion. In animal studies, the combination of subcutaneous and intravenous administration of antivenom was no better than intravenous administration alone[42].

Skin testing is not necessary or recommended prior to administration of the current antivenom [7,43]. In addition to cleavage and removal of the immunogenic Fc portion of the immunoglobulin molecule, the currently available antivenom undergoes column affinity purification. Symptoms of acute anaphylactoid reactions, such as pruritus, urticaria, or wheezing occur in approximately 6% of patients [37,44]. Most cases are mild and do not preclude continued administration of antivenom. However, severe acute allergic reactions, including reactions involving airway compromise, have been described [37,45]. As a result, the panel recommends that the first dose of antivenom be administered in a clinical setting, such as an emergency department or intensive care unit, where the medications, equipment, and skilled personnel required to manage an airway emergency are immediately available. If there is no acute reaction to initial dosing, subsequent doses of antivenom can be administered in a less monitored setting, such as a hospital ward. Management of allergic effects is discussed below.

The panel recommended increasing the initial dose of antivenom to 8 to 12 vials in patients who present with immediately life-threatening venom effects, such as shock or serious active bleeding. In a large Phase IV study of severely envenomated pit viper victims (approximately 13% of the patients who were treated with antivenom), 69% of patients required more than one dose of antivenom to achieve initial control [37]. The median dose of antivenom used to achieve initial control in this population was 9 vials (interquartile range: 6 to 15 vials). Additionally, bites by large rattlesnakes are associated with more severe envenomation that requires administration of higher doses of antivenom [46]. In the presence of immediately life-threatening venom effects, the panel believed that the benefit of more rapid control of hypotension and bleeding expected with an aggressive dosing strategy exceeded the benefit that could be gained by administration of a more typical 4 to 6 vial antivenom dose in patients. Although this practice is common among the panel members, it has not been empirically studied.

The panel recommends routine administration of intravenous crystalloid solution to any pit viper victim who requires antivenom. Venom causes vasodilatation and capillary leakage, leading to relative volume depletion, and antivenom infusion can cause histamine release. Although the standard dilution of antivenom is one dose (4 - 6 vials) in 250 ml normal saline, some panelists choose large volumes of dilution (1000 ml) in patients for whom there is no contraindication. In general, each dose of antivenom is infused over one hour. Faster infusion may be preferred for critically ill patients who are in shock or actively hemorrhaging. Some panelists start antivenom administration at a slow initial rate (e.g. 25 ml/hr for 10 minutes), followed by an increased infusion rate (balance of dose administered over 50 minutes) if no acute hypersensitivity reaction is observed, while others prefer a single infusion rate strategy to reduce medical errors. In the absence of data, the panel did not make an infusion rate recommendation. Although routine pre-treatment with antihistamines is not generally recommended, some panelists do so as a matter of clinical routine. No evidence bears on this practice.

Because antivenom is intended to neutralize the dose of injected venom, the pediatric dose of antivenom is the same as the adult dose. Although this hypothesis has not been critically tested, it is consistent with observation in pediatric case series [47,48].

### Assessment for initial control of the envenomation syndrome (boxes 5 and 11)

Approximately half of antivenom-treated patients require more than one dose of antivenom to achieve initial control [11]. Therefore, the treating physician should examine the patient and repeat indicated laboratory studies soon after antivenom is administered to evaluate for treatment response. Because fibrinogen and platelet levels change rapidly after antivenom administration, coagulation studies and platelet counts should be rechecked within one hour of antivenom dosing. If initial control of the envenomation syndrome is achieved, the patient can be observed, either as an inpatient or in a clinical observation unit, to make certain that this clinical response is maintained. If the first dose of antivenom does not succeed in producing initial control, the initial dose should be repeated. Failure to achieve initial control after two doses of antivenom is uncommon. In a large retrospective study, only 17% of rattlesnake victims and 2% of *Agkistrodon *(copperhead and water moccasin) victims required more than 12 vials of antivenom to achieve initial control, and the presence of thrombocytopenia and neurologic venom effects prior to antivenom therapy were independently associated with the difficulty achieving initial control [41,49]. Consultation with a physician, clinical toxicologist, or other expert who has specific training and expertise in the management of venomous snakebite is recommended in this and other high-risk clinical situations. Information about how to reach such an expert can be found on the algorithm (box 12), or below.

### Post-stabilization monitoring and administration of maintenance therapy (boxes 6 and 13)

Snake envenomation is a dynamic clinical process. Although clinical improvement virtually always follows administration of adequate antivenom doses, recurrence or delayed-onset of one or more venom effects occurs in approximately half of patients treated with Fab antivenom [11]. Serial physician examinations and laboratory studies are necessary to detect recurrent or delayed-onset venom effects. When it occurs, local tissue recurrence typically develops within 6 to 36 hours of initial control. Recurrent local tissue effects are clinically evident to the patient and generally respond well to re-treatment with antivenom. The onset of recurrent or delayed-onset hematologic venom effects is much more variable, with most cases occurring 2 - 7 days after initial control and some cases up to 10 days after initial control [25,36]. When antivenom is administered to treat recurrent coagulopathy or thrombocytopenia, the treatment response is generally attenuated compared with the response to initial antivenom therapy [26,28,30,31,50-52]. Hematologic venom effects are most often clinically occult; few patients experience medically significant bleeding even in the setting of profound defibrination or thrombocytopenia [26].

The ideal duration of hospitalization and frequency of follow-up observations and laboratory studies is unknown. After the first 24 hours, the marginal benefit of continued hospitalization appears to be small, and follow-up monitoring in the outpatient setting is appropriate for most patients. The panel recommends that patients be observed in the hospital for 18 - 24 hours following initial control of the envenomation syndrome, with serial examinations performed approximately every 6 - 8 hours during this interval. The panel recommends that most patients have laboratory studies (protime, hemoglobin, platelet count, and fibrinogen level) measured twice prior to discharge: once 6 - 12 hours after initial control, which appears to be the time at which the risk of recurrent hematologic venom effects is greatest, and again prior to discharge [25]. Unfavourable trends in protime, fibrinogen, or platelet counts should prompt additional testing. Because only 5 - 10% of copperhead envenomation victims develop hematologic venom effects at any time, it is reasonable to forego one set of follow-up lab tests in those copperhead victims who have never manifest coagulopathy, thrombocytopenia, or systemic bleeding [40].

In the current FDA-approved prescribing information, the manufacturer of antivenom recommends administration of additional 2-vial doses of antivenom given 6, 12, and 18 hours after initial control is achieved [43]. In a randomized clinical trial, this practice reduced the proportion of patients with recurrent local tissue effects from 8/16 (50%) to 0/15 (0%) [11]. However, cases of recurrent local tissue effects developing in maintenance-treated patients have been reported [26,39,40,47]. The cost-effectiveness of maintenance therapy is unclear; in a randomized clinical trial, patients randomized to receive maintenance therapy and patients randomized to receive additional antivenom administered as needed to treat recurrent swelling received the same median number of antivenom vials [11]. The extent to which maintenance therapy reduces the risk of recurrent and delayed-onset hemorrhagic venom effects is not precisely known. Results of the antivenom phase III premarketing trial appeared to show a reduction in the incidence rate of recurrent hematologic venom effects in patients who received maintenance therapy. In that trial, recurrent thrombocytopenia was noted in 2/14 (14%) patients who received maintenance antivenom therapy, compared with 9/16 (56%) patients who did not receive maintenance therapy [43]. In the same study, recurrent hypofibrinogenemia was noted in 2/14 (14%) of patients receiving maintenance therapy and 7/16 (44%) of those who did not receive maintenance. Small sample size and the large proportion of patients in the no-maintenance group who received rescue therapy due to recurrent local tissue effects makes any estimate of the effect of maintenance antivenom therapy difficult to interpret. Furthermore, the baseline risk of hematologic venom effects varies approximately 10-fold based on the envenomating species and the initial severity of the envenomation, with rattlesnake victims and patients with high initial clinical severity apparently at the greatest risk [26,36,40]. In a recent case series from central Arizona, 32% of patients developed new or recurrent hematologic abnormalities after initial control [31]. Finally, the panelists noted that the safety of a "watchful waiting" strategy is wholly dependent on the quality and frequency of follow-up observations, which may vary depending on local hospital resources and staffing patterns.

In light of the above information, the practice of administering maintenance antivenom therapy is controversial. Historically, some centers recommend maintenance therapy universally, while others do so in the minority of cases [26,39,40]. Given the wide variation in clinical practice patterns the panelists concluded that a "one size fits all" or simplified decision rule was inappropriate for the question of whether to administer maintenance therapy. The panel recommended consultation with a regional poison center or local snakebite treatment expert to assist in the determination of whether to give maintenance antivenom therapy.

### Management of patients with apparent dry bites or minor envenomations (boxes 9 and 10)

**Figure 2:**
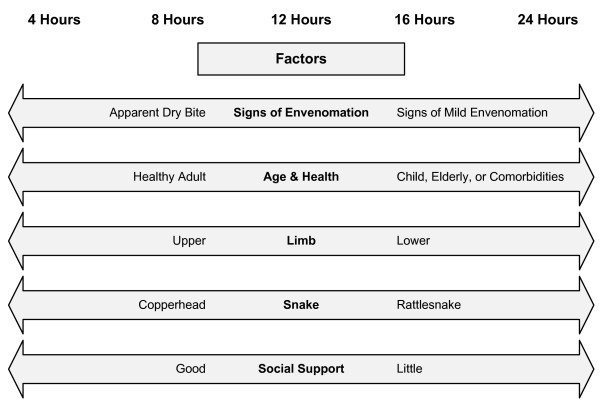
**Factors Influencing Observation Time for Patients with Apparent Dry Bites and Initially Minor Envenomations Managed Without Antivenom**.

Approximately 20 - 25% of crotaline snakebites are "dry"; no venom effects develop [18]. Although the majority of patients with apparent dry bites have not been envenomated, some patients who initially present with a wound but no other signs of envenomation (i.e. no swelling, ecchymosis, vesicle formation, or hematologic or systemic venom effects) develop signs of envenomation after a latent period of minutes to hours [53]. In addition, some patients present with apparent minor venom effects (ecchymosis, swelling, and/or vesicles limited to the immediate area of the bite site without systemic venom effects). All panel members reported having treated patients who presented in this manner and subsequently developed significant progressive signs of envenomation. To our knowledge, there are no data to describe the typical time course or define a "safe" period of observation after which the risk of delayed-onset venom effects is minimal, although the best available evidence suggests that 6 hours is not long enough in many cases [53]. Cost-benefit implications are largely unknown. The panel members recommended that, in general, patients with apparent non-envenomation be observed in a health care facility for at least 8 hours, with repeat platelet count, prothrombin time, fibrinogen, and hemoglobin measurement prior to discharge. Anecdotal evidence suggests some patients, such as children and those with lower extremity envenomations, may develop significant tissue effects more than 8 hours after an apparent dry bite, and therefore may require longer observation. Patients with apparently minor envenomation and no evidence of progression should be observed longer, in the range of 12 - 24 hours. These observation periods might be appropriately shortened or prolonged based on a number of factors, including the age and general health of the patient, bite location, envenomating species, social support available to the patient, patient preference, and ability of the treating facility to provide efficient and cost-effective observation services.(Figure 2) Patients who develop no venom effects during the observation period should be discharged with instructions to return promptly if signs of envenomation develop or progress.

### Discharge criteria and post-discharge management (boxes 7, 8, and 14)

Patients who have had no further progression of venom effects during an appropriate period of observation may be discharged when certain criteria are met. As with any patient going home from the hospital, the patient must be able to perform activities of daily living unassisted or with the assistance available in the home, have adequate pain control on oral medications, and have no other outstanding medical issues requiring hospital care. In addition, the patient should not have any unfavorable trends in protime, fibrinogen levels, or platelet counts, since deterioration in one or more of these parameters may be an early sign of recurrent or delayed-onset hematologic venom effects.

Following discharge, patients should be instructed to maintain limb elevation as much as possible to speed resolution of swelling. Progressive swelling that does not improve with elevation or signs of abnormal bleeding, such as gingival bleeding, easy bruising, or melena, may be the hallmark of recurrent hematologic venom effects, and should lead to prompt re-evaluation.

Serum sickness, a type III hypersensitivity reaction caused by administration of exogenous proteins, is a known complication of antivenom therapy. In prospective studies, approximately 5 - 10% of patients treated with ovine Fab antivenom develop signs of serum sickness, such as fever, rash, myalgias, and arthralgias [44]. Serum sickness following Fab antivenom administration is generally mild and responds well to treatment with oral antihistamines and corticosteroids. At the time of discharge, patients should be instructed about the symptoms of serum sickness and given directions regarding follow-up care should serum sickness develop.

Few data exist to inform the number and timing of follow-up visits. In general, the panel felt that mandatory follow-up visits were not needed for patients who had minimal envenomation and did not require antivenom administration. Similarly, because the risk of late hematologic venom effects is small, routine follow-up of patients with uncomplicated copperhead snake envenomations who did not develop hematologic venom effects during hospitalization is unlikely to provide clinical benefit to a patient. On the other hand, patients with rattlesnake envenomations and those who demonstrated hematologic venom effects during the acute phase of therapy are at high risk for late hematologic venom effects that most often occur 2 - 7 days after antivenom therapy [25-30,51,54,55]. Follow-up visits with laboratory testing are therefore recommended 2 - 3 days and 5 - 7 days after discharge, with additional visits as needed based on signs, symptoms, and laboratory trends.

### High risk situations requiring expert consultation (box 12)

Of the crotaline victims treated with antivenom, approximately 13% develop severe envenomation [37]. Clinicians who practice outside of referral centers that see a large volume of snakebite patients rarely have the opportunity to develop a large base of experience treating critically envenomated patients. For this reason, the panel identified certain high-risk clinical situations in which consultation with a physician who has specific training and expertise in the management of crotaline snakebite is strongly encouraged. In institutions where bedside consultation is available, bedside consultation should be sought. In the remainder of institutions, telephone consultation, facilitated by a regional poison center, is recommended. Even if local practice calls for transfer of patients from the presenting facility to a tertiary care center, early consultation with a physician-expert (or, alternatively, a pharmacologist or clinical toxicologist with specific training and expertise in snakebite management) is recommended to ensure that early interventions are ideal and all appropriate preparations are made at the receiving facility.

#### Patients with life-threatening envenomation

Frank hypotension, active hemorrhage, and airway edema are uncommon but life-threatening manifestations of crotaline snakebite [37]. Evidence supports a benefit from antivenom therapy in the former two situations, while the use of antivenom combined with active airway management is recommended for the latter situation based on case reports [26,36,37,56]. For the reasons previously described, the panel recommended a larger initial dose of antivenom for patients with shock or active hemorrhage due to snakebite. Only 1% of snake envenomations involve the head or neck, but the experience of panel members suggests a high risk of subsequent loss of airway. This situation is considered analogous to thermal airway burns, in which early endotracheal intubation may prevent the need for surgical airway placement and its attendant complications.

#### Difficult to control envenomations

Even in a severely envenomated cohort, initial control of the envenomation syndrome can be achieved with one or two doses of antivenom in most patients [37]. Case reports of refractory neurotoxicity and hematologic toxicity exist, but the available evidence do not define a point at which further administration of antivenom is likely to be futile [26,33,37,50]. In addition to assisting with cost-benefit estimation, a physician-expert may be able to identify secondary complications (e.g. rhabdomyolysis from persistent myokymia) that require additional interventions. Consultation with a physician-expert is recommended in cases where initial control of the envenomation syndrome has not been achieved following two doses of antivenom.

#### Recurrent or delayed-onset of venom effects

As described above, the management of recurrent or delayed-onset hematologic venom effects is controversial. Most patients tolerate hematologic venom effects well, but several serious cases and one fatality have been described [27]. Compared to the initial treatment response, the response to repeat antivenom dosing is often attenuated and may be transient [26,28,50,52]. While guidelines exist, there is no settled clinical decision rule for which patients require retreatment, and estimates of which patients are at highest risk are largely derived from experience with other diseases [57]. Although the risk to the patient of additional antivenom dosing appears to be minimal, cost-benefit considerations are significant, particularly when re-hospitalization is required. For these reasons, the panel recommends direct consultation with a physician-expert to assist in management of these patients.

#### Allergic reactions to antivenom

Signs of immediate hypersensitivity to antivenom are observed in 5 - 6% of patients treated with ovine Fab antivenom [37,44]. Although most of these reactions are relatively minor and do not preclude antivenom therapy, some are severe. As described above, the initial management of a hypersensitivity reaction is straightforward: halt the antivenom infusion and administer antihistamines, corticosteroids, and fluids as needed until signs of hypersensitivity have resolved. Epinephrine may be required for severe reactions. At this point, the decision to resume or discontinue antivenom therapy involves a complex balancing of risk and benefit that the panel could not reduce to an algorithm. Because few clinicians have the opportunity to gain experience with this uncommon clinical scenario, consultation with an expert clinician is recommended.

#### Hematologic venom effects when transfusion is considered

Thrombin-like enzymes in crotaline venom incompletely cleave fibrinogen, leading to the formation of an unstable fibrin clot that is not cross-linked [23,58]. The mechanism that underlies venom-induced thrombocytopenia is less well-understood; venom-induced injury to platelet cell membranes and endothelial activation caused by microvascular damage have been proposed [24,58,59]. Transfusion alone can produce transient improvement in coagulation parameters and platelet counts, but rarely has a sustained effect unless adequate doses of antivenom have also been administered. Aggressive antivenom administration should always precede fresh frozen plasma, cryoprecipitate, or platelet transfusion if antivenom is available. Transfusion is indicated for cases in which medically significant bleeding is occurring. Whether administration of additional antivenom and transfusion are appropriate for patients who are not actively bleeding but who have profound coagulopathy, profound thrombocytopenia, or multicomponent hematological venom effects (both thrombocytopenia and defibrinogenation) remains unclear [60]. Although most patients tolerate hematological venom effects without incident, severe or fatal bleeding events have occurred [27-31]. Transfusion also has associated cost and risks. Consultation prior to transfusion is recommended, when possible, to maximize the utility of transfusion and reduce unnecessary use of blood products.

#### Rhabdomyolysis

Although crotaline venom is directly myotoxic, clinically severe rhabdomyolysis is uncommon in the United States [61]. Although routine creatine kinase measurement is not recommended, specific patients, such as those with severe local tissue injury and/or prolonged systemic neurotoxicity can develop rhabdomyolysis. Consultation with a physician-expert is recommended in these cases.

#### Suspected compartment syndrome

Crotaline snakebite can produce pain, swelling, induration, paresthesias, color changes (e.g. bluish discoloration from bruising), difficult-to-palpate pulses, and tenderness in the envenomated extremity, mimicking the initial signs of compartment syndrome. However, true compartment syndrome is much less common, and a prospective observational study in humans showed that most rattlesnake victims have greater blood flow in the envenomated than in the non-envenomated limb [62]. Animal research and human experience demonstrate that antivenom administration reduces compartment pressures, and surgical groups who used to perform fasciotomy frequently now acknowledge that antivenom administration often precludes the need for fasciotomy [9,40,63,64]. In one large case series of patients treated in a tertiary referral center, only 8/236 (3.4%) of patients received a fasciotomy or digital dermotomy [10]. Measurement of compartment pressure prior to consideration of fasciotomy is recommended. Compartment pressure measurement may not be feasible in cases of digital envenomation. Consultation with a physician-expert is recommended whenever compartment syndrome is suspected and prior to any fasciotomy or digit dermotomy.

#### Venom-induced hives and angioedema

Anaphylactic and anaphylactoid reactions to venom are uncommon manifestations of snakebite which can range in severity from urticarial rash to multisystem organ failure and angioedema causing airway loss [65]. At least 2 deaths have been reported [66,67]. Although standard therapy includes antihistamines, steroids, epinephrine, and antivenom, the ideal management of this condition is unknown. Because these patients are often critically ill and require aggressive, multimodal therapy, panel members recommended expert consultation.

#### Complicated wound issues

Crotaline envenomation causes local tissue necrosis by a variety of mechanisms, some of which are not reversible with antivenom therapy [68]. Although venom-induced inflammation often mimics infection, true bacterial cellulitis is uncommon, affecting approximately 3% of snakebite patients [69]. Rarely, severe infections have been reported [70]. Confusion about whether an envenomated extremity is inflamed or infected may lead to unnecessary medical care, including intravenous antibiotics and prolonged hospitalization [71]. Decisions about debridement and tissue grafting may also be complex. Consultation with an expert who has experience managing envenomated wounds may improve these decisions.

### Treatments to avoid in pit viper snakebite (box 15)

The panel recommends against several therapies that are commonly utilized to treat crotaline envenomation, but which are ineffective, unnecessary, or harmful. Wound incision and suction does not remove meaningful amounts of venom and can worsen local tissue injury [72,73]. Although little evidence exists to condemn the topical application of ice, this measure appears to be ineffective [74]. More aggressive forms of cryotherapy, such as ice water immersion, have been associated with severe iatrogenic tissue injury [75]. Although this issue has not been subjected to study, panel members recommended avoiding the use of non-steroidal anti-inflammatory drugs (NSAIDs) because of the theoretical harm associated with the platelet dysfunction caused by NSAIDs in a potentially thrombocytopenic patient. Prophylactic antibiotics, prophylactic fasciotomy, and the routine use of blood products should be avoided for the reasons discussed above. Application of electrical current from a spark plug or hand-held "stun gun" has been recommended for therapy based on anecdotal experience from a missionary physician in Ecuador[76]. Subsequent animal research and human experience have shown this practice to be ineffective and associated with significant tissue injury [77-81]. There is a paucity of data about the role of corticosteroids in crotaline snakebite. Based on unpublished experience and controlled trial data from the United States showing that corticosteroids do not improve outcome in old world viper (family *Viperidae*, subfamily *Viperidae*) envenomation, administration of corticosteroids is reserved for treatment of hypersensitivity phenomena [82,83].

Although data from envenomations by snakes native to the United States are lacking, arterial tourniquet application is ineffective and sometimes associated with apparent harm when used to treat South American crotaline snakes [84]. Although pressure immobilization has a confirmed role in the management of highly neurotoxic elapid snake envenomations, its role in crotaline envenomation is unclear. In porcine models of severe western diamondback rattlesnake envenomation, pressure immobilization prolonged survival, with varying effects on local tissue injury [85,86]. Similarly, lymphatic constricting bands reduce the absorption of venom into the systemic circulation in animal models, but whether this strategy is more likely to improve or worsen overall outcomes is unknown [87]. Neither pressure immobilization nor use of lymphatic constricting bands is recommended.

## Discussion

Management of a simple case of crotaline snakebite involves many clinical decisions. Clinical trials in this area are challenging to conduct. To our knowledge, only five clinical trials of crotaline snakebite have been performed. One of these was randomized [11]. A second randomized trial was attempted, but terminated early due to low enrollment [88]. A third identified trial was non-randomized [12]. Finally, two trials were identified involving an antivenom product that is not currently licensed in the US. One of these trials has been completed, but results have only been published in preliminary form [89]. The other is ongoing [90].

In situations where high quality evidence does not exist, clinical recommendations can be primarily influenced by factors other than the results of clinical trials. These factors include uncertainty in the estimates of likely benefit, risk, inconvenience, and cost of therapy, and varying values of clinicians and patients [91]. Available techniques for evidence-based decision-making do not provide tools for dealing with regional variations in disease characteristics, differences in treatment resources available at different centers, or situations in which the amount of unpublished experience equals or exceeds the amount of data in the peer-reviewed literature. By definition, evidence-based hypothesis testing cannot begin until each specific clinical question is defined; this creates a circular problem when creating complex, highly-branched treatment algorithms. For these reasons, we believed that an evidence-informed structured consensus process would produce a final result that was more useful to clinicians and patients than a formal evidence-based medicine approach. Notwithstanding these limitations, it is possible to describe these treatment recommendations in GRADE terms [91]. The decision to give antivenom to patients with limb-threatening envenomation or severe systemic effects is a strong recommendation based on moderate quality evidence; despite the lack of placebo-controlled trials, concordant results of a large number of observational studies and animal experiments make it clear that the benefits of therapy outweigh the associated risks and burdens. All other recommendations are weak recommendations based on very low quality evidence.

This process, and its output, have limitations. Although we took care to minimize the introduction of commercial bias through conflict-of-interest disclosure, exclusion of the project sponsor from the decision-making process, diversity of panel membership, use of a trained facilitator, and structured decision-making methods, we cannot exclude the possibility that prior relationships between project participants and the manufacturer of antivenom may have influenced the opinions and practice patterns of panel members. These concerns may be mitigated somewhat by the observation that, although the treatment algorithm contained here is more comprehensive than recently published treatment guidance from unrelated authors, the indications for antivenom are essentially similar [9,92,93].

Rather than rely solely on expert opinion, we utilized several strategies to inform the decision-making process. We performed a comprehensive literature review and made all publications containing original data available at the time of panel deliberations. In addition, we utilized our gap analysis to identify data needs and develop information targeted to those needs. To this end, we performed focused analysis of line-level data collected in the phase II and III clinical trials, a phase IV database created by the antivenom manufacturer, and a separate prospectively-collected database from a high-volume snakebite treatment center. Whenever the above methods did not produce clear data to inform a treatment decision, we explicitly acknowledged this limitation in the manuscript.

## Conclusions

Venomous snakebite is a complex and dynamic clinical entity that is characterized by a wide variation in clinical effects and response to therapy. Using a structured, evidence-informed decision-making process, we provide treatment guidelines that may reduce unnecessary variation in care and improve clinical outcomes.

## Competing interests

SPB is an employee of Faculty Medical Group of Loma Linda University School of Medicine, which has received research funding from Protherics. SPB derives no personal financial benefit from this relationship.

EJL and RCD are employees of the Denver Health and Hospital Authority, which has received research funding from Protherics. None of these authors derive personal financial benefit from this relationship.

WB, VB, JNB, WPK, WHR, AMR, SAS, and DAT declare that they have no competing interests.

The views expressed by VB and DAT in this article are those of the authors, and do not reflect the official policy or position of the US Air Force, the US Navy, the US Department of Defense, or the US government.

## Authors' contributions

EJL conceived the project. EJL and RCD secured funding. EJL drafted the initial version of the treatment algorithm. EJL, AMR, SPB, SAS, and staff of the Rocky Mountain Poison and Drug Center prepared data analyses for presentation at the meeting. WB, VK, JNB, SPB, WPK, WHR, AMR, SAS, DAT, and RCD were voting members of the expert consensus panel, which was chaired by a professional facilitator. SCC provided input during algorithm development and participated in the expert consensus panel as a non-voting member. EJL created the manuscript draft. All authors read, revised and contributed to the final manuscript. EJL takes responsibility for the work as a whole.

## Pre-publication history

The pre-publication history for this paper can be accessed here:

http://www.biomedcentral.com/1471-227X/11/2/prepub

## References

[B1] O'Neil ME Mack KA Gilchrist J Wozniak EJ Snakebite injuries treated in United States emergency departments, 2001-2004 Wilderness Environ Med 2007 18 4 281 287 18076294 10.1580/06-WEME-OR-080R1.1

[B2] Langley R Animal-related fatalities in the United States - an update Wilderness Environ Med 2005 16 67 74 15974255 10.1580/1080-6032(2005)16[67:afitus]2.0.co;2

[B3] Spiller HA Bosse GM Ryan ML Use of antivenom for snakebites reported to United States poison centers Am J Emerg Med 2010 in press 20837254 10.1016/j.ajem.2009.03.021

[B4] Walter FG Stolz U Shirazi F McNally J Walter FG Stolz U Shirazi F McNally J Epidemiology of severe and fatal rattlesnake bites published in the American Association of Poison Control Centers' Annual Reports Clin Toxicol 2009 47 7 663 669 10.1080/15563650903113701 19640239

[B5] Goto CS Feng SY Crotalidae polyvalent immune Fab for the treatment of pediatric crotaline envenomation Pediatr Emerg Care 2009 25 4 273 279 10.1097/PEC.0b013e31819f1f1e 19369845

[B6] Campbell BT Corsi JM Boneti C Jackson RJ Smith SD Kokoska ER Pediatric snakebites: lessons learned from 114 cases J Pediatr Surg 2008 43 7 1338 41 10.1016/j.jpedsurg.2007.11.011 18639692

[B7] Gold BS Dart RC Barish RA Bites of venomous snakes N Engl J Med 2002 347 5 347 356 10.1056/NEJMra013477 12151473

[B8] Dart RC Can steel heal a compartment syndrome caused by rattlesnake venom? Ann Emerg Med 2004 44 2 105 107 10.1016/j.annemergmed.2004.03.013 15278080

[B9] Corneille MG Larson S Stewart RM Dent D Myers JG Lopez PP McFarland MJ Cohn SM A large single-center experience with treatment of patients with crotalid envenomations: outcomes with and evolution of antivenin therapy Am J Surg 2006 192 6 848 852 10.1016/j.amjsurg.2006.08.056 17161106

[B10] Tanen D Ruha A Graeme K Curry S Epidemiology and hospital course of rattlesnake envenomations cared for at a tertiary referral center in central Arizona Acad Emerg Med 2001 8 2 177 182 10.1111/j.1553-2712.2001.tb01284.x 11157295

[B11] Dart RC Seifert SA Boyer LV Clark RF Hall E McKinney P McNally J Kitchens CS Curry SC Bogdan GM A randomized multicenter trial of *Crotalinae *polyvalent immune Fab (ovine) antivenom for the treatment for crotaline snakebite in the United States Arch Intern Med 2001 161 16 2030 2036 10.1001/archinte.161.16.2030 11525706

[B12] Dart RC Seifert SA Carroll L Clark RF Hall E Boyer-Hassen LV Curry SC Kitchens CS Garcia RA Affinity-purified, mixed monospecific crotalid antivenom ovine Fab for the treatment of crotalid venom poisoning Ann Emerg Med 1997 30 1 33 39 10.1016/S0196-0644(97)70107-0 9209222

[B13] Stanford CF Bebarta VS Holstege CP Bush SP Richardson WH Olsen D Dart RC Is Crotaline Fab Antivenom efficacious for severe envenomations? [abstract] Clin Toxicol 2007 45 6 619

[B14] Suankratay C Wilde H Nunthapisud P Khantipong M Suankratay C Wilde H Nunthapisud P Khantipong M Tetanus after white-lipped green pit viper (*Trimeresurus albolabris*) bite Wilderness Environ Med 2002 13 4 256 261 12510783 10.1580/1080-6032(2002)013[0256:tawlgp]2.0.co;2

[B15] Habib A Tetanus complicating snakebite in northern Nigeria; clinical presentation and public health implications Acta Trop 2003 85 1 87 91 10.1016/S0001-706X(02)00234-6 12505187

[B16] Anonymous ACIP Recommendations for Vaccination 2010 Atlanta, Georgia: Centers for Disease Prevention and Control http://www.cdc.gov/vaccines/pubs/acip-list.htm accessed July 1, 2010

[B17] Russell F Snake Venom Poisoning 1983 3 Great Neck, NY, Scholium International

[B18] Curry S Horning D Brady P Requa R Kunkel D Vance M The legitimacy of rattlesnake bites in central Arizona Ann Emerg Med 1989 18 6 658 663 10.1016/S0196-0644(89)80523-2 2729691

[B19] Ownby CL Structure, function and biophysical aspects of the myotoxins from snake venoms J Toxicol - Toxin Rev 1998 17 2 213 238

[B20] Gutierrez JM Rucavado A Snake venom metalloproteinases: Their role in the pathogenesis of local tissue damage Biochimie 2000 82 9-10 841 850 10.1016/S0300-9084(00)01163-9 11086214

[B21] Moura-da-Silva A Laing G Paine M Dennison J Politi V Crampton J Theakston D Processing of pro-tumour necrosis factor-α by venom metalloproteinases: a hypothesis towards explaining local tissue damage following snakebite Toxicon 1997 35 6 814 815 10.1016/S0041-0101(97)90330-5 8814237

[B22] Crocker P Zad O Milling T Maxson T King B Whorton E Human cytokine response to Texas crotaline envenomation before and after antivenom administration Am J Emerg Med 2010 in press 10.1016/j.ajem.2009.04.038 20887908

[B23] Kitchens CS Hemostatic aspects of envenomation by North American snakes Hematol Oncol Clin North Am 1992 6 5 1189 1195 1400081

[B24] Hutton R Warrell D Action of snake venom components on the haemostatic system Blood Rev 1993 7 3 176 189 10.1016/0268-960X(93)90004-N 8241832

[B25] Boyer LV Seifert SA Clark RF McNally JT Williams SR Nordt SP Walter FG Dart RC Recurrent and persistent coagulopathy following pit viper envenomation Arch Intern Med 1999 159 7 706 710 10.1001/archinte.159.7.706 10218750

[B26] Ruha AM Curry SC Beuhler M Katz K Brooks DE Graeme KA Wallace K Gerkin R Lovecchio F Wax P Selden B Initial postmarketing experience with Crotalidae polyvalent immune Fab for treatment of rattlesnake envenomation Ann Emerg Med 2002 39 6 609 615 10.1067/mem.2002.123698 12023703

[B27] Kitchens C Eskin T Fatality in a case of envenomation by *Crotalus adamanteus *initially successfully treated with polyvalent ovine antivenom followed by recurrence of defibrinogenation syndrome J Med Toxicol 2008 4 3 180 183 10.1007/BF03161198 18821492 PMC3550043

[B28] Fazelat J Teperman SH Touger M Fazelat J Teperman SH Touger M Recurrent hemorrhage after western diamondback rattlesnake envenomation treated with Crotalidae polyvalent immune Fab (ovine) Clin Toxicol 2008 46 9 823 826 10.1080/15563650701753849 18608290

[B29] O'Brien NF DeMott MC Suchard JR Clark RF Peterson BM Recurrent coagulopathy with delayed significant bleeding after crotaline envenomation Pediatr Emerg Care 2009 25 7 457 459 19606002 10.1097/PEC.0b013e3181ab7871

[B30] Camilleri C Offerman S Gosselin R Albertson T Camilleri C Offerman S Gosselin R Albertson T Conservative management of delayed, multicomponent coagulopathy following rattlesnake envenomation Clin Toxicol 2005 43 3 201 206 15902796

[B31] Ruha AM Curry SC Albrecht C Riley B Pizon A Late hematologic toxicity following treatment of rattlesnake envenomation with crotalidae polyvalent immune fab antivenom Toxicon 2010 57 1 53 9 10.1016/j.toxicon.2010.09.014 20920516

[B32] Bush SP Siedenburg E Neurotoxicity associated with suspected southern Pacific rattlesnake (*Crotalus viridis helleri*) envenomation Wilderness Environ Med 1999 10 4 247 249 10628285 10.1580/1080-6032(1999)010[0247:nawssp]2.3.co;2

[B33] Richardson WH Goto CS Gutglass DJ Williams SR Clark RF Rattlesnake envenomation with neurotoxicity refractory to treatment with crotaline Fab antivenom Clin Toxicol 2007 45 5 472 475 10.1080/15563650701338187 17503249

[B34] Clark RF Williams SR Nordt SP Boyer-Hassen LV Successful treatment of crotalid-induced neurotoxicity with new polyspecific crotalid Fab antivenom Ann Emerg Med 1997 30 1 54 57 10.1016/S0196-0644(97)70111-2 9209226

[B35] Glenn JL Straight RC Wolfe MC D L Geographical variation in *Crotalus scutulatus scutulatus *(Mojave rattlesnake) venom properties Toxicon 1983 21 1 119 130 10.1016/0041-0101(83)90055-7 6342208

[B36] Lavonas EJ Schaefer TH Kokko J Mlynarchek SL Bogdan GM Crotaline Fab antivenom appears to be effective in cases of severe North American pit viper envenomation: an integrative review BMC Emerg Med 2009 9 13 epub 19545426 10.1186/1471-227X-9-13 PMC2713980

[B37] Lavonas EJ Kokko J Schaeffer TH Mlynarchek SL Bogdan GM Dart RC Short-term outcomes following Fab antivenom therapy for severe crotaline snakebite Ann Emerg Med 2010 57 2 128 137 10.1016/j.annemergmed.2010.06.550 20952098

[B38] Lavonas EJ Kerns WP Gerardo CJ Richardson W Whitlow KS Berkoff DJ Long-term limb function outcomes after copperhead snakebite [abstract] Ann Emerg Med 2008 52 4 S141 142 10.1016/j.annemergmed.2008.06.354

[B39] Bush SP Green SM Moynihan JA Hayes WK Cardwell MD Crotalidae polyvalent immune Fab (ovine) antivenom is efficacious for envenomations by Southern Pacific rattlesnakes (*Crotalus helleri*) Ann Emerg Med 2002 40 6 619 624 10.1067/mem.2002.129939 12447339

[B40] Lavonas EJ Gerardo CJ O'Malley G Arnold TC Bush SP Banner W Steffens M Kerns WP Initial experience with Crotalidae polyvalent immune Fab (ovine) antivenom in the treatment of copperhead snakebite Ann Emerg Med 2004 43 2 200 206 10.1016/j.annemergmed.2003.08.009 14747809

[B41] Yin S Kokko J Lavonas E Mlynarchek S Bogdan G Schaeffer T Factors associated with difficulty achieving initial control with Crotalidae Polyvalent Immune Fab antivenom in snakebite patients Acad Emerg Med 2010 18 1 46 52 10.1111/j.1553-2712.2010.00958.x 21166732

[B42] Offerman S Barry J Richardson W Tong T Tanen D Bush S Clark R Subcutaneous crotaline Fab antivenom for the treatment of rattlesnake envenomation in a porcine model Clin Toxicol 2009 47 1 61 68 10.1080/15563650701750613 19153852

[B43] Anonymous Prescribing Information: CroFab^®^ http://www.btgplc.com/PubContent/Docs/CroFab%20PI.pdf accessed July 1, 2010

[B44] Cannon R Ruha AM Kashani J Acute hypersensitivity reactions associated with administration of Crotalidae polyvalent immune Fab antivenom Ann Emerg Med 2008 51 4 407 411 10.1016/j.annemergmed.2007.09.036 18191286

[B45] Holstege CP Wu J Baer AB Immediate hypersensitivity reaction associated with the rapid infusion of Crotalidae polyvalent immune Fab (ovine) Ann Emerg Med 2002 39 6 677 679 10.1067/mem.2002.124442 12023715

[B46] Janes D Bush S Kolluru G Large snake size suggests increased snakebite severity in patients bitten by rattlesnakes in southern California Wilderness Environ Med 2010 21 2 120 126 10.1016/j.wem.2010.01.010 20591373

[B47] Offerman SR Bush SP Moynihan JA Clark RF Offerman SR Bush SP Moynihan JA Clark RF Crotaline Fab antivenom for the treatment of children with rattlesnake envenomation Pediatrics 2002 110 5 968 971 10.1542/peds.110.5.968 12415038

[B48] Schmidt JM Schmidt JM Antivenom therapy for snakebites in children: is there evidence? Curr Opin Pediatr 2005 17 2 234 238 10.1097/01.mop.0000152621.67049.f2 15800419

[B49] Chuang R Kokko J Mlynarchek S Schaeffer T Bogdan G Lavonas E Seifert SA Rattlesnake versus *Agkistrodon *envenomations: venom effects differences [abstract] Abstracts from Venom Week 2009, June 1-4, 2009 Albuquerque NM. J Med Toxicol 2010 6 2 240 1

[B50] Offerman SR Barry JD Schneir A Clark RF Biphasic rattlesnake venom-induced thrombocytopenia J Emerg Med 2003 24 3 289 293 10.1016/S0736-4679(02)00763-1 12676300

[B51] Lintner C Keyler D Bilden E Seifert SA Prairie rattlesnake (*Crotalus viridis viridis*) envenomation: recurrent coagulopathy in a child treated with immune Fab [abstract] Snakebites in the new millennium. Proceedings of a state-of-the-art symposium. J Med Toxicol 2006 2 1 33 4

[B52] Seifert SA Boyer LV Dart RC Porter RS Sjostrom L Relationship of venom effects to venom antigen and antivenom serum concentrations in a patient with *Crotalus atrox *envenomation treated with a Fab antivenom Ann Emerg Med 1997 30 1 49 53 10.1016/S0196-0644(97)70110-0 9209225

[B53] Hurlbut K Dart R Spaite D McNally J Reliability of clinical presentation for predicting significant pit viper envenomation Ann Emerg Med 1988 17 4 438 439

[B54] Miller MA Dyer JE Olson KR Two cases of rattlesnake envenomation with delayed coagulopathy Ann Emerg Med 2002 39 3 348 10.1067/mem.2002.121998 11867998

[B55] Wasserberger J Ordog G Merkin TE Southern Pacific rattlesnake bite: a unique clinical challenge J Emerg Med 2006 31 3 263 266 10.1016/j.jemermed.2005.09.018 16982358

[B56] Kerns W Tomaszewski C Airway obstruction following canebrake rattlesnake envenomation J Emerg Med 2001 20 4 377 380 10.1016/S0736-4679(01)00315-8 11348818

[B57] Boyer LV Seifert SA Cain JS Recurrence phenomena after immunoglobulin therapy for snake envenomations: Part 2. Guidelines for clinical management with crotaline Fab antivenom Ann Emerg Med 2001 37 2 196 201 10.1067/mem.2001.113134 11174239

[B58] Ruha AM Curry SC Recombinant factor VIIa for treatment of gastrointestinal hemorrhage following rattlesnake envenomation Wilderness Environ Med 2009 20 2 156 160 10.1580/08-WEME-CR-229R1.1 19594209

[B59] Gutierrez JM Rucavado A Escalante T Diaz C Hemorrhage induced by snake venom metalloproteinases: Biochemical and biophysical mechanisms involved in microvessel damage Toxicon 2005 45 8 997 1011 10.1016/j.toxicon.2005.02.029 15922771

[B60] Yip L Rational use of Crotalidae polyvalent immune Fab (ovine) in the management of crotaline bite Ann Emerg Med 2002 39 6 648 650 10.1067/mem.2002.124450 12023708

[B61] Kitchens C Hunter S Van Mierop L Severe myonecrosis in a fatal case of envenomation by the canebrake rattlesnake (*Crotalus horridus atricaudatus*) Toxicon 1987 25 4 455 458 10.1016/0041-0101(87)90080-8 3617084

[B62] Curry S Kraner J Kunkel D Ryan P Vance M Requa R Ruggieri S Noninvasive vascular studies in management of rattlesnake envenomations to extremities Ann Emerg Med 1985 14 11 1081 1084 10.1016/S0196-0644(85)80926-4 4051274

[B63] Tanen D Danish D Grice G Riffenburgh R Clark R Fasciotomy worsens the amount of myonecrosis in a porcine model of crotaline envenomation Emerg Med 2004 44 2 99 104 10.1016/j.annemergmed.2004.01.009 15278079

[B64] Shaw BA Hosalkar HS Rattlesnake bites in children: Antivenin treatment and surgical indications J Bone Joint Surg 2002 84 9 1624 1629 12208920

[B65] Curry S O'Connor A Ruha A Rapid-onset shock and/or anaphylactoid reactions from rattlesnake bites in central Arizona Clin Toxicol 2010 in press

[B66] Litovitz T Klein-Schwartz W Rodgers G Cobaugh D Youniss J Omslaer J May M Woolf A Benson B 2001 Annual report of the American Association of Poison Control Centers Toxic Exposure Surveillance System Am J Emerg Med 2002 20 5 391 452 10.1053/ajem.2002.34955 12216043

[B67] Lai M Klein-Schwartz W Rodgers G Abrams J Haber D Bronstein A Wruk K 2005 Annual report of the American Association of Poison Control Centers' National Poisoning and Exposure Database Clin Toxicol 2006 44 803 932 10.1080/15563650600907165 17015284

[B68] Ownby C Colberg T Odell G *In vivo *ability of antimyotoxin a serum plus polyvalent (*Crotalidae*) antivenom to neutralize prairie rattlesnake (*Crotalus viridis viridi*s) venom Toxicon 1986 24 2 197 200 10.1016/0041-0101(86)90122-4 3705096

[B69] Clark RF Selden BS Furbee B The incidence of wound infection following crotalid envenomation J Emerg Med 1993 11 5 583 586 10.1016/0736-4679(93)90313-V 8308237

[B70] Angel MF Zhang F Jones M Henderson J Chapman SW Angel MF Zhang F Jones M Henderson J Chapman SW Necrotizing fasciitis of the upper extremity resulting from a water moccasin bite South Med J 2002 95 9 1090 1094 12356121

[B71] Scharman EJ Noffsinger VD Copperhead snakebites: clinical severity of local effects Ann Emerg Med 2001 38 1 55 61 10.1067/mem.2001.116148 11423813

[B72] Alberts MB Shalit M LoGalbo F Suction for venomous snakebite: a study of "mock venom" extraction in a human model Ann Emerg Med 2004 43 2 181 186 10.1016/S0196-0644(03)00813-8 14747805

[B73] Bush SP Hegewald KG Green SM Cardwell MD Hayes WK Effects of a negative pressure venom extraction device (Extractor) on local tissue injury after artificial rattlesnake envenomation in a porcine model Wilderness Environ Med 2000 11 3 180 8 11055564 10.1580/1080-6032(2000)011[0180:eoanpv]2.3.co;2

[B74] Cohen W Wetzel W Kadish A Local heat and cold application after eastern cottonmouth moccasin (*Agkistrodon piscivorus*) envenomation in the rat: effect on tissue injury Toxicon 1992 30 11 1383 1386 10.1016/0041-0101(92)90513-5 1485335

[B75] Frank H What are we doing? An evaluation of cryotherapy for envenomation Western J Med 1971 114 5 25 27 PMC1501983 5087879

[B76] Guderian R Mackenzie C Williams J High voltage shock treatment for snake bite Lancet 1986 2 229 10.1016/S0140-6736(86)92535-3 2873481

[B77] Dart RC Gustafson RA Failure of electric shock treatment for rattlesnake envenomation Ann Emerg Med 1991 20 6 659 661 10.1016/S0196-0644(05)82389-3 2039106

[B78] Johnson E Kardong KV Mackessy SP Electric shocks are ineffective in the treatment of lethal effects of rattlesnake envenomation in mice Toxicon 1987 25 12 1347 1349 10.1016/0041-0101(87)90013-4 3438923

[B79] Howe NR Meisenheimer JL Electric shock does not save snakebitten rats Ann Emerg Med 1988 17 3 254 256 10.1016/S0196-0644(88)80118-5 3257850

[B80] Welch B Gales BJ Use of stun guns for venomous bites and stings: a review Wilderness Environ Med 2001 12 2 111 117 11434486 10.1580/1080-6032(2001)012[0111:uosgfv]2.0.co;2

[B81] Gold BS Electric shock: a potentially hazardous approach to treating venomous snakebite Maryland Med J 1993 42 3 244 245 8350681

[B82] Reid H Thean P Martin W Specific antivenin and prednisone in viper-bite poisoning: controlled trial BMJ 1963 2 5369 1387 1380 10.1136/bmj.2.5369.1378 14063030 PMC1873581

[B83] Reid H Theakston R The management of snake bite Bull World Health Organ 1983 61 6 885 895 6609008 PMC2536242

[B84] Amaral CFS Campolina D Dias MB Bueno CM Rezende NA Tourniquet ineffectiveness to reduce the severity of envenoming after *Crotalus durissus *snake bite in Belo Horizonte, Minas Gerais, Brazil Toxicon 1998 36 5 805 808 10.1016/S0041-0101(97)00132-3 9655642

[B85] Bush S Green S Laack T Hayes W Cardwell M Tanen D Pressure immobilization delays mortality and increases intracompartmental pressure after artificial intramuscular rattlesnake envenomation in a porcine model Ann Emerg Med 2004 44 6 599 604 10.1016/j.annemergmed.2004.06.007 15573035

[B86] Meggs W Courtney C O'Rourke D Brewer K Pilot studies of pressure-immobilization bandages for rattlesnake envenomations Clin Toxicol 2010 48 1 61 63 10.3109/15563650903376071 19888893

[B87] Burgess J Dart R Egen N Mayersohn M Effects of constriction bands on rattlesnake venom absorption: A pharmacokinetic study Ann Emerg Med 1992 21 9 1086 1093 10.1016/S0196-0644(05)80649-3 1514719

[B88] Anonymous The efficacy of Crotaline Fab antivenom for copperhead snake envenomations - clinical trial [Clinical trial identifier NCT00303303] http://www.clinicaltrials.gov/ct2/show/NCT00868309 accessed July 1, 2010

[B89] Anonymous A comparison of *Crotalinae *(pit viper) equine immune F(ab)2 antivenom (Anavip) and Crotalidae polyvalent immune Fab, ovine antivenom (CroFab) in the treatment of pit viper envenomation [Clinical trial identifier NCT00868309] http://www.clinicaltrials.gov/ct2/results?term = 00868309 accessed July 1, 2010

[B90] Anonymous Phase 3 multicenter comparative study to confirm safety and effectiveness of the F(ab)2 antivenom Anavip [Clinical trial identifier NCT00636116] http://www.clinicaltrials.gov/ct2/results?term=NCT00636116 accessed July 1, 2010

[B91] Guyatt G Oxman A Kunz R Falck-Yttr Y Vist G Liberati A Schunemann H Rating quality of evidence and strength of recommendations: Going from evidence to recommendations BMJ 2008 336 1049 1051 10.1136/bmj.39493.646875.AE 18436948 PMC2335261

[B92] Weant K Johnson P Bowers R Armistead J Evidence-based, multidisciplinary approach to the development of a Crotalidae polyvalent antivenin (CroFab) protocol at a university hospital Ann Pharmacother 2010 44 447 455 10.1345/aph.1M527 20124465

[B93] Cribari C Management of poisonous snakebite 2004 Chicago: American College of Surgeons Committee on Trauma http://www.facs.org/trauma/publications/snakebite.pdf accessed July 1, 2010

